# Supplementary material for: The North American bullfrog draft genome provides insight into hormonal regulation of long noncoding RNA
Source: Nat Commun. 2017 Nov 10;8:1433. doi: 10.1038/s41467-017-01316-7 (PMC5681567; doi:10.1038/s41467-017-01316-7)
Supplement: Supplementary file 1 — Supplementary Information [file 41467_2017_1316_MOESM1_ESM.pdf]

Supplementary Information

Supplementary Figures

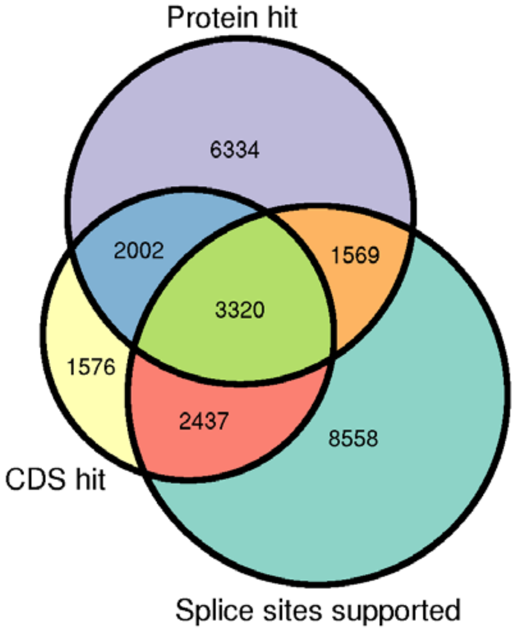

Supplementary Figure 1. Selection criteria for the high confidence gene set. Transcripts were included in the high confidence set if they satisfied one or more of the following criteria: 1) the gene contained at least one splice site, and all splice sites were confirmed by an alignment to external transcript evidence (splice sites supported); 2) the CDS had a BLASTn alignment to a BART contig with at least 95% identity along 99% of its length (CDS hit); 3) the protein sequence encoded by the CDS had a BLASTp alignment to a human or amphibian Swiss-Prot protein sequence with at least 50% identity along 90% of its length (Protein hit).

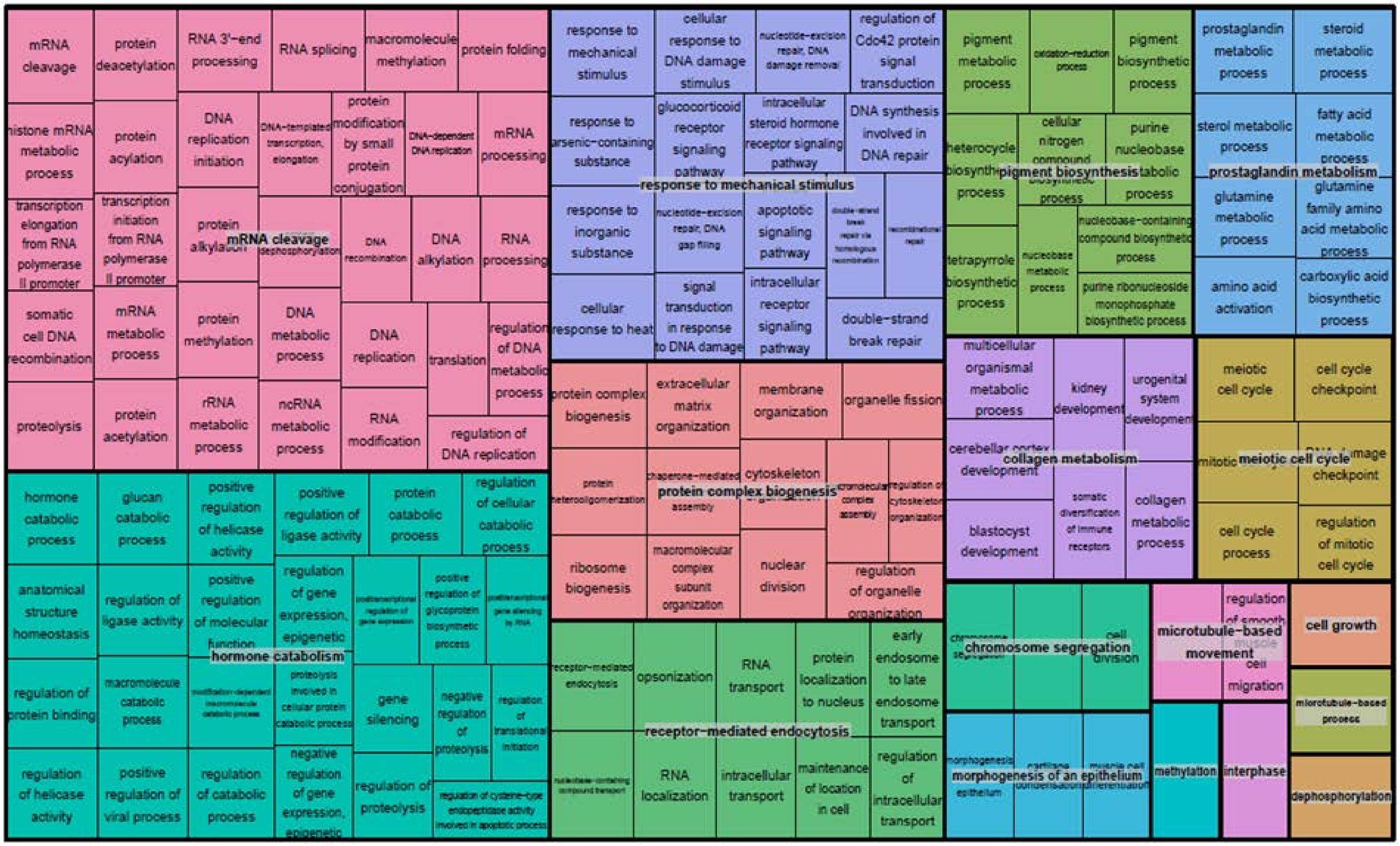

Supplementary Figure 2. Enriched GO terms associated with genes differentially expressed in *R. catesbeiana* back skin following exposure to 10 nM T3 for 48 h. RNA-Seq reads were aligned to the genome with STAR, read counts per high-confidence transcript determines with HTSeq, and differential expression in the T3 treated group relative to the vehicle control determined using DESeq2 , where significance was at the 0.05 level.

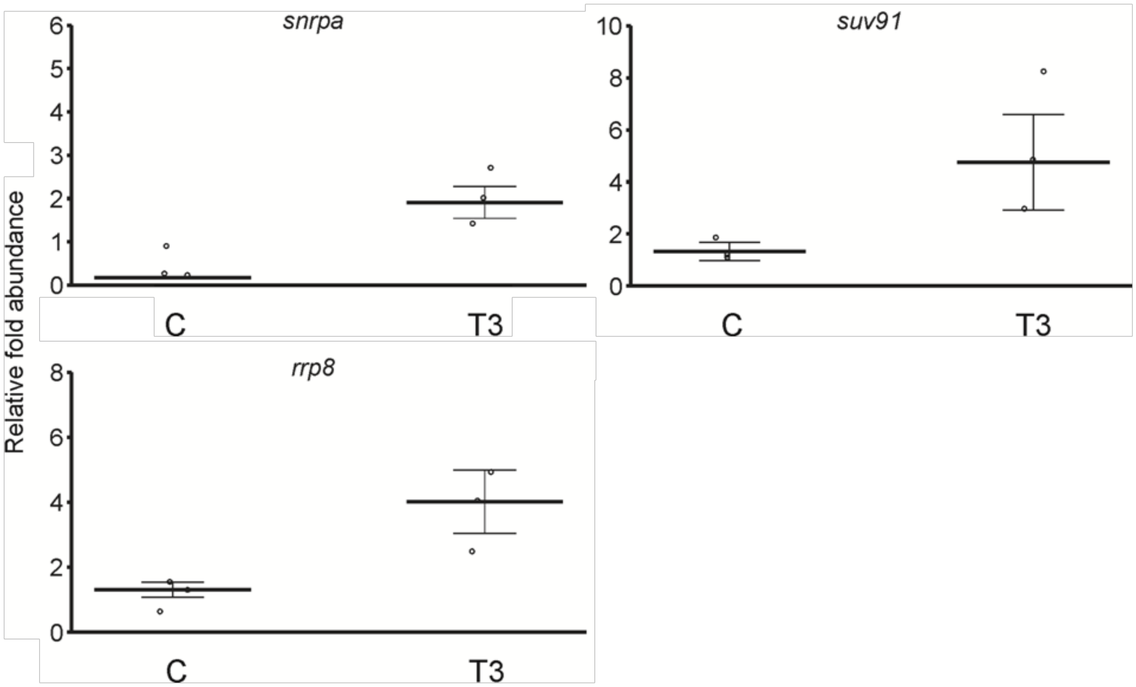

Supplementary Figure 3. qPCR analysis of select transcripts encoding proteins involved in RNA/DNA processing in the back skin. Premetamorphic tadpoles (n = 3 per treatment) were injected with 10 pmol/g body weight of T3 or dilute sodium hydroxide solvent (C) and the back skin collected after 48 h for RNA isolation and qPCR analysis. The median fold abundance of transcripts encoding U1 small nuclear ribonucleoprotein A (*snrpa*), ribosomal RNA processing protein 8 (*rrp8*), and histone-lysine-N-methyltransferase *(suv91*) relative to the control is shown. Whiskers indicate the median absolute deviation, and the open circles denote the fold difference of individual animals. All transcripts were significantly different (Mann-Whitney U test, $p < 0.05$).

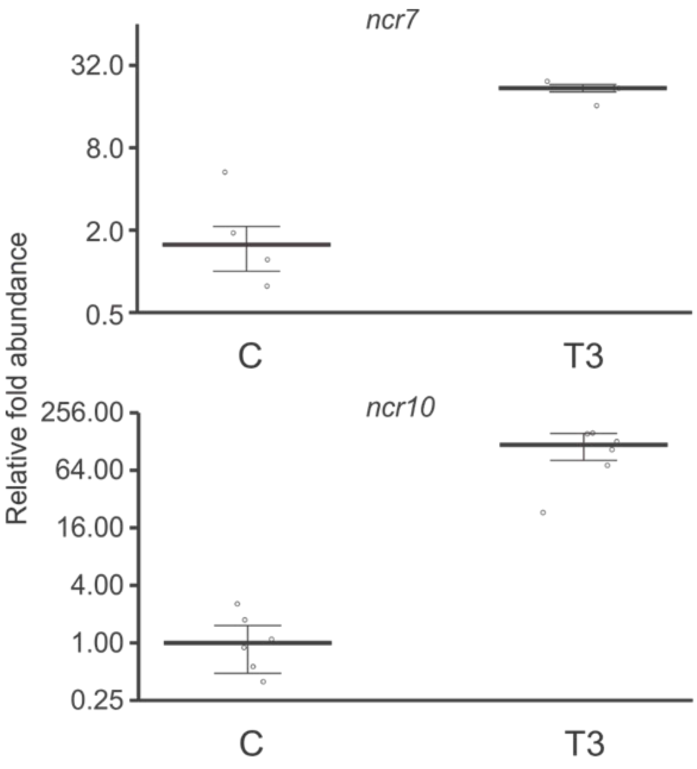

Supplementary Figure 4. qPCR analysis of select lncRNA transcripts in the back skin. Premetamorphic tadpoles (n = 6 per treatment) were injected with 10 pmol/g body weight of T3 or dilute sodium hydroxide solvent (C) and the back skin collected after 48 h for RNA isolation and qPCR analysis. The median fold abundance of transcripts of candidate lncRNAs, *ncr7* and *ncr10* relative to the control is shown. Whiskers indicate the median absolute deviation, and the open circles denote the fold difference of individual animals. Both transcripts were significantly different (Mann-Whitney U test, $p < 0.05$).

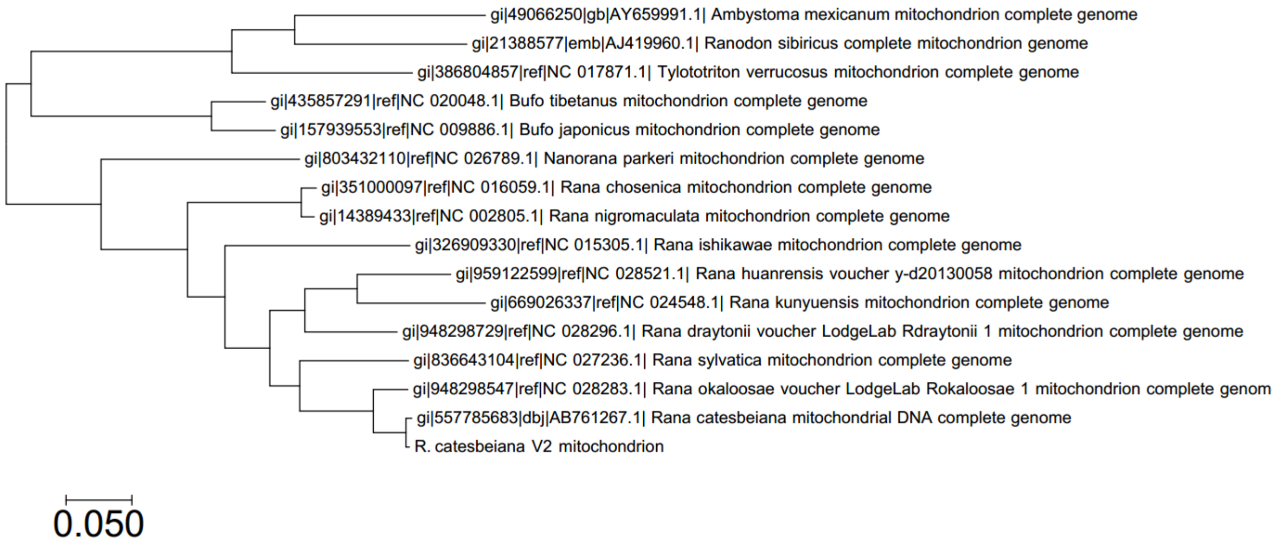

Supplementary Figure 5. Molecular phylogenetic analysis of complete mitochondrial genomes of selected amphibians by Maximum Likelihood method. The evolutionary history was inferred by using the Maximum Likelihood method based on the Tamura-Nei model[24]. The tree with the highest log likelihood (-91034.06) is shown. Initial tree(s) for the heuristic search were obtained automatically by applying Neighbor-Join and BioNJ algorithms to a matrix of pairwise distances estimated using the Maximum Composite Likelihood (MCL) approach, and then selecting the topology with superior log likelihood value. The tree is drawn to scale, with branch lengths measured in the number of substitutions per site. The analysis involved 16 nucleotide sequences (see Supplementary Table 8). All positions containing gaps and missing data were eliminated. There were a total of 10,646 positions in the final dataset. Evolutionary analyses were conducted in MEGA7[25].

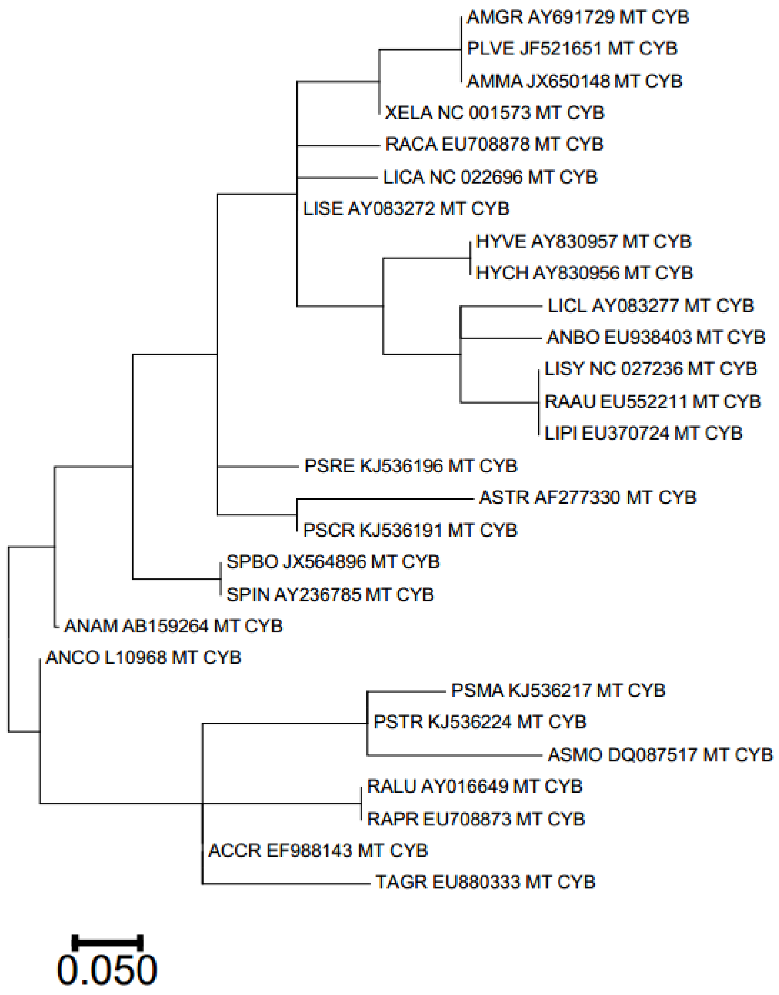

Supplementary Figure 6. Molecular phylogenetic analysis of mitochondrial *cyb* (MT CYB) genes of selected amphibians by Maximum Likelihood method. Sequences are annotated with the first two letters of the organisms' genus and species, respectively, followed by the NCBI GenBank accession number. See Supplementary Figure 5 legend for details of analysis and Supplementary Table 9 for additional information including the species code.

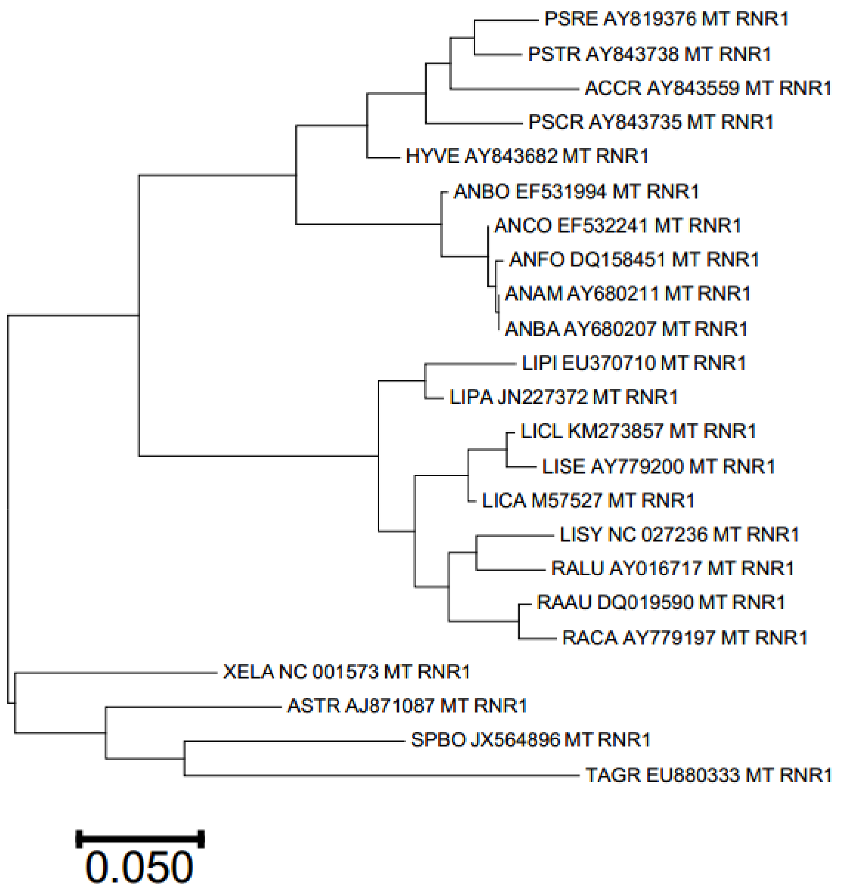

Supplementary Figure 7. Molecular phylogenetic analysis of mitochondrial *rnr1* (MT RNR1) genes of selected amphibians by Maximum Likelihood method. Sequences are annotated with the first two letters of the organisms' genus and species, respectively, followed by the NCBI GenBank accession number. See Supplementary Figure 5 legend for details of analysis and Supplementary Table 9 for additional information including the species code.

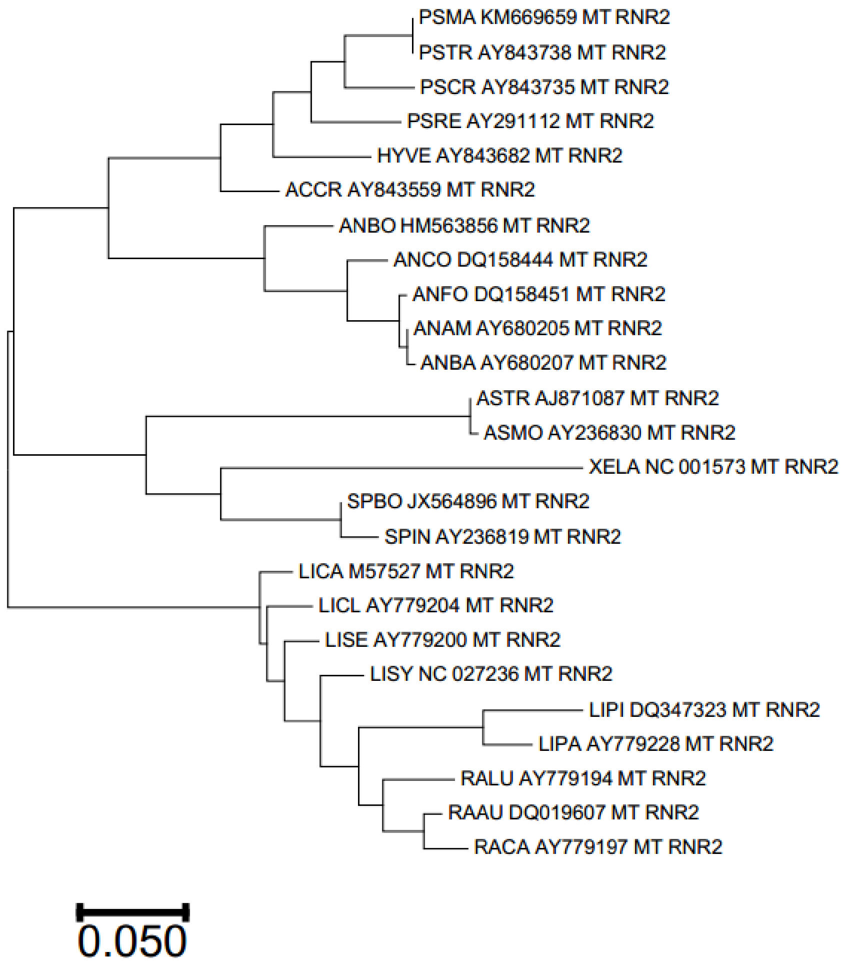

Supplementary Figure 8. Molecular phylogenetic analysis of mitochondrial *rnr2* (MT RNR2) genes of selected amphibians by Maximum Likelihood method. Sequences are annotated with the first two letters of the organisms' genus and species, respectively, followed by the NCBI GenBank accession number. See Supplementary Figure 5 legend for details of analysis and Supplementary Table 9 for additional information including the species code.

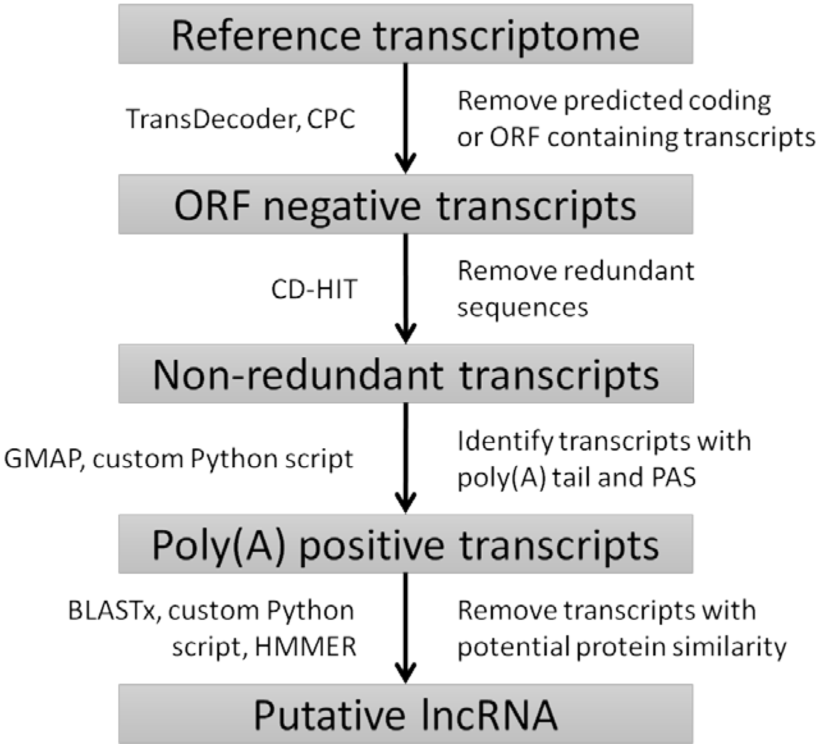

Supplementary Figure 9. Workflow for detection of putative lncRNA transcripts. BART contigs with low protein coding potential were excluded, as were redundant sequences. Polyadenylated transcript sequences were selected, and any residual sequences that may have encoded a peptide sequence with similarity to any database sequences were eliminated to arrive at the set of putative lncRNA sequences.

Supplementary Tables

Supplementary Table 1. Scaffolding the North American bullfrog genome with long-range distance information. TGA = Targeted Gene Assembly; WGA = Whole Genome Assembly.

Methodology	Data Source	Number of merges	NG50 (bp)	BUSCO Complete	BUSCO Complete + Fragmented
ABySS v1.9.0 k160	MPET (7kbp)	NA	23,361	1169	2146
RAILS v0.1	SLR (Moleculo)	56,784	30,085	1282	2276
ABySS - longscaffolding v1.9.0	Kollector TGA BART	NA	33,847	1497	2413
LINKS v1.7 x10	SLR (Moleculo)	29,178	34,492	1500	2435
LINKS v1.7	MPET (7 kbp)	108,578	50,123	1646	2539
LINKS v1.7 x7	Kollector TGA and k128 WGA	77,885	58,021	1749	2623
ARCS	Chromium linked reads	15,059	68,964	1787	2650

Supplementary Table 2. Estimated proportion of repetitive DNA sequences in *R. catesbeiana* (version 2) and select organisms.

Species	Approx. haploid genome size (Gbp)	Estimated interspersed repeat content (%)	Reference
Rana (Lithobates) catesbeiana	5.8	62	The present study
Homo sapiens	3.1	56	Smit *et al.* (2013)[4]
Nanorana parkeri	2.3	47	Sun *et al.* (2015)[26]
Xenopus tropicalis	1.5	43	Sun *et al.* (2015)[26]

Supplementary Table 3. Comparison of relative fold abundance of select back skin transcripts significantly increased upon T3 exposure.

	Fold abundance relative to control	
Transcript	RNA-seq	qPCR
thrb	3.1 ± 0.1	8.4 ± 0.1*
RNA/DNA processing		
snrpa	5.2 ± 0.2	11.1 ± 2.2
rrp8	3.5 ± 0.2	3.1 ± 0.8
suv39h1	2.5 ± 0.2	3.6 ± 1.4

* From Maher *et al.* (2016)[3]

Supplementary Table 4. Targeted qPCR primer information.

Gene transcript	Primer name	Primer sequence	Amplicon length (bp)	Annealing Temperature (^{o}C)
snrpa	150110	TCCCAGAAGAGACAAACGAG	211	64
	150111	GCAGGCTACTTTTTGGCAA		
rrp8	150114	TGACTCTGCGTTCCCGTAT	254	64
	150115	AGCATCACCACAGCCAAA		
suv91	150116	AAATGCGGATTACTACTG	248	60
	150117	CTCCAAATGAGTTAGGGT		
ncr7	160157	GTTCATCAAGTAGGTCTCCAAT	254	60
	160158	TATCACCAGTCAGAGCCATAA		
ncr10	160141	ACAAGTAAGGACAGGGAGTGG	244	60
	160142	GGAGTCAGGGTTCTGTAGG		

Supplementary Table 5. *R. catesbeiana* RNA-Seq data. Reads are available under NCBI BioProject PRJNA286013. DE = read sets used for the differential gene expression experiment; BART = read sets assembled with Trans-ABySS to construct BART. References: (1) Hammond *et al.* (2015); (2) the present study.

Tissue	Chemical Condition	Sequencing Platform	Read Length (bp)	Read Pairs (M)	Utilization	Reference
Back Skin	dilute NaOH	HiSeq2000	75	139	BART	(1)
Back Skin	dilute NaOH	HiSeq2000	75	90	BART	(1)
Back Skin	dilute NaOH	HiSeq2500	100	135	DE, BART	(2)
Back Skin	dilute NaOH	HiSeq2500	100	178	DE, BART	(2)
Back Skin	dilute NaOH	HiSeq2500	100	156	DE, BART	(2)
Back Skin	10 nM T_3	HiSeq2000	75	121	BART	(1)
Back Skin	10 nM T_3	HiSeq2000	75	136	BART	(1)
Back Skin	10 nM T_3	HiSeq2500	100	158	DE, BART	(2)
Back Skin	10 nM T_3	HiSeq2500	100	141	DE, BART	(2)
Back Skin	10 nM T_3	HiSeq2500	100	161	DE, BART	(2)
Tail Fin	dilute NaOH	HiSeq2000	75	96	BART	(1)
Tail Fin	dilute NaOH	HiSeq2000	75	101	BART	(1)
Tail Fin	10 nM T_3	HiSeq2000	75	193	BART	(1)
Tail Fin	10 nM T_3	HiSeq2000	75	122	BART	(1)
Lung	dilute NaOH	HiSeq2000	75	108	BART	(1)
Lung	dilute NaOH	HiSeq2000	75	114	BART	(1)
Lung	10 nM T_3	HiSeq2000	75	125	BART	(1)
Lung	10 nM T_3	HiSeq2000	75	115	BART	(1)
Brain	dilute NaOH	HiSeq2000	75	110	BART	(1)
Brain	dilute NaOH	HiSeq2000	75	100	BART	(1)
Brain	dilute NaOH	HiSeq2000	75	98	BART	(1)
Brain	10 nM T_3	HiSeq2000	75	116	BART	(1)
Brain	10 nM T_3	HiSeq2000	75	101	BART	(1)
Brain	10 nM T_3	HiSeq2000	75	126	BART	(1)
Olfactory Bulb	solvent	MiSeq	100	9	BART	Unpublished
Olfactory Bulb	solvent	MiSeq	100	14	BART	Unpublished
Olfactory Bulb	solvent	MiSeq	100	8	BART	Unpublished
Olfactory Bulb	solvent	MiSeq	100	8	BART	Unpublished
Olfactory Bulb	Chemical Cocktail	MiSeq	100	12	BART	Unpublished
Olfactory Bulb	Chemical Cocktail	MiSeq	100	11	BART	Unpublished
Olfactory Bulb	Chemical Cocktail	MiSeq	100	8	BART	Unpublished
Olfactory Bulb	Chemical Cocktail	MiSeq	100	9	BART	Unpublished

Supplementary Table 6. DNA poly(A) hexamer motifs considered for detection of cleavage site. Observed frequency of usage in *Homo sapiens* noted for reference.

DNA hexamer	Usage frequency (*Homo sapiens*, %)*
AATAAA	52.0%
ATTAAA	14.9%
TATAAA	3.2%
AGTAAA	2.7%
AATATA	1.7%
CATAAA	1.3%
GATAAA	1.3%
AATACA	1.2%
TTTAAA	1.2%
AAGAAA	1.1%
AAAAAG	0.8%
AATGAA	0.8%
AATAGA	0.7%
ACTAAA	0.6%
AAAACA	0.5%
GGGGCT	0.3%

* From Beaudoing *et al.* (2000)[27]

Supplementary Table 7. Amphibian species included in the CATSA database.

Species or genus	TSA size (Mbp)
Ambystoma mexicanum	4.2
Bufotes viridis	45
Hynobius chinensis	97
Hynobius retardus	445
Leptobrachium boringii	45
Megophrys	45
Microhyla fissipes	85
Odorrana margaretae	41
Pelophylax nigromaculatus	47
Polypedates megacephalus	53
Pseudacris (Hyliola) regilla	36
Rana (Lithobates) clamitans	37
Rana (Lithobates) pipiens	886
Rhacophorus dennysi	53
Rhacophorus omeimontis	39
Tylototriton wenxianensis	87

Supplementary Table 8. Complete mitochondrial genome sequences used in conjunction with our assembled *R. catesbeiana* mitochondrial genome sequence in the phylogenetic analysis.

Species	GenBank Accession
Ambystoma mexicanum	AY659991.1
Bufo japonicas	NC_009886.1
Bufo tibetanus	NC_020048.1
Nanorana parkeri	NC_026789.1
Rana (Lithobates) catesbeiana	AB761267.1
Rana chosenica	NC_016059.1
Rana draytonii	NC_028296.1
Rana huanrensis	NC_028521.1
Rana ishikawae	NC_015305.1
Rana kunyuensis	NC_024548.1
Rana nigromaculata	NC_002805.1
Rana (Lithobates) okaloosae	NC_028283.1
Ranodon sibiricus	AJ419960.1
Rana (Lithobates) sylvatica	NC_027236.1
Tylototriton verrucosus	NC_017871.1

Supplementary Table 9. Mitochondrial genes used in phylogenetic analysis.

	GenBank Accession		
Species (Code)	*cyb*	*rnr1*	*rnr2*
Acris crepitans (ACCR)	EF988143	AY843559	AY843559
Anaxyrus americanus (ANAM)	AB159264	AY680211	AY680205
Anaxyrus baxteri (ANBA)	x	AY680207	AY680207
Anaxyrus boreas (ANBO)	EU938403	EF531994	HM563856
Anaxyrus cognatus (ANCO)	L10968	EF532241	DQ158444
Anaxyrus fowleri (ANFO)	x	DQ158451	DQ158451
Ambystoma gracile (AMGR)	AY691729	x	x
Ambystoma macrodactylum (AMMA)	JX650148	x	x
Ascaphus montanus (ASMO)	DQ087517	x	AY236830
Ascaphus truei (ANTR)	AF277330	AJ871087	AJ871087
Hyla chrysoscelis (HYCH)	AY830956	x	x
Hyla versicolor (HYVE)	AY830957	AY843682	AY843682
Pseudacris crucifer (PSCR)	KJ536191	AY843735	AY843735
Pseudacris maculate (PSMA)	KJ536217	x	KM669659
Pseudacris (Hyliola) regilla (PSRE)	KJ536196	AY819376	AY291112
Pseudacris triseriata (PSTR)	KJ536224	AY843738	AY843738
Plethodon vehiculum (PLVE)	JF521651	x	x
Rana aurora (RAAU)	EU552211	DQ019590	DQ019607
Rana cascadae (RACA)	EU708878	AY779197	AY779197
Rana (Lithobates) catesbeiana (LICA)	NC022696	M57527	M57527
Rana (Lithobates) clamitans (LICL)	AY083277	KM273857	AY779204
Rana luteiventris (RALU)	AY016649	AY016717	AY779194
Rana (Lithobates) palustris (LIPA)	x	JN227372	AY779228
Rana (Lithobates) pipiens (LIPI)	EU370724	EU370710	DQ347323
Rana pretiosa (RAPR)	EU708873	x	x
Rana (Lithobates) septentrionales (LISE)	AY083272	AY779200	AY779201
Rana (Lithobates) sylvatica (LISY)	NC027236	NC027236	NC027236
Spea bombifrons (SPBO)	JX564896	JX564896	JX564896
Spea intermontana (SPIN)	AY236785	x	AY236819
Taricha granulosa (TAGR)	EU880333	EU880333	x
Xenopus laevis (XELA)	NC001573	NC001573	NC001573

Supplementary Table 10. ABySS-Bloom sequence identity calculations between certain mammalian genome assemblies and the *Homo sapiens* genome.

		Estimated time since divergence (MYA)		
		Homo sapiens	*Rattus norvegicus*	*Oryctolagus. cuniculus*
Estimated identity (%)	*Homo sapiens*		90	90
	Rattus norvegicus	81.0 +/- $2.4x10^{-3}$		82
	Oryctolagus cuniculus	83.1 +/- $4.4x10^{-4}$	80.6 ± 1.37 x 10^{-3}	

Supplementary Methods

Targeted gene assembly with Kollector

Kollector is an alignment-free targeted *de novo* assembly pipeline that uses thousands of transcript sequences concurrently to inform the localized assembly of corresponding gene loci[1]. Kollector scans whole genome shotgun sequencing data to recruit reads that have sequence similarity to input transcripts or previously recruited reads, which are then assembled with ABySS. This greedy approach to read collection enables resolution of intronic regions for the assembly of complete genes.

To provide long-distance information for scaffolding, we used Kollector to reconstruct the gene loci of the transcripts contained in the BART reference transcriptome. The BART transcripts were randomly divided into 80 bins of approximately 10,000 transcripts each, and Kollector ran on each bin in parallel (-j 12 -s 0.9 -r 128 -k 128). To evaluate success of the targeted gene assemblies (TGA), the input transcripts were aligned to the Kollector-assembled sequences with BLASTn[2], and those transcripts that aligned with 90% sequence identity and 90% query coverage were considered to have had their corresponding gene successfully reconstructed. Transcripts that did not meet these criteria were re-binned and re-tried in the next iteration with parameters tuned for higher sensitivity. This is achieved by lowering the r parameter (number of nucleotide matches required for recruiting a read) and the value of k used in the assembly step. After 5 Kollector iterations (k and r = 128, 112, 96, 80, 64), 78% of BART transcripts were successfully assembled according to our criteria.

Protein coding gene prediction

Prediction of protein coding genes was performed using the MAKER genome annotation pipeline[3] (version 2.31.8). This framework included RepeatMasker[4] to mask repetitive sequence

elements based on the core RepBase repeat library[5]. Augustus[6], SNAP[7] and GeneMark[8] were also run within the MAKER2 pipeline to produce *ab initio* gene predictions. BLASTx[2], BLASTn[2], and exonerate[9] alignments of human and amphibian Swiss-Prot protein sequences[10] (retrieved 16 February 2016) and BART were combined with the gene predictions to yield the gene models. MAKER2 was first applied to an early version of the bullfrog genome assembly, and the 1000 best gene models by eAED score were used for retraining SNAP[11].

Gene ontology and pathway analysis

Due to the particularly extensive biological information available for human proteins, a second round of BLASTp alignments were performed between the high confidence set of predicted proteins and the Swiss-Prot human proteins, using the same alignment thresholds noted above. The Uniprot accession IDs and log fold-changes of the differentially expressed genes were collected, input to the Ingenuity Pathway Analysis tool (Qiagen Bioinformatics, Redwood City, CA), and its core analysis was run with default settings. The Database for Annotation, Visualization and Integrated Discovery (DAVID)[12] v6.8 was also used with default settings to perform gene annotation enrichment analysis on the differentially expressed genes versus the background of all bullfrog genes with Uniprot annotations. The enriched annotations were visualized with ReviGO[13] with default settings.

Assembly versioning

The bullfrog genome project produced three main assemblies to date, predominantly differentiated by the incorporation of additional sequencing reads (version 2) and the utilization of progressively more sequence data for scaffolding (version 2 and 3). Version 1 used the 150 bp HiSeq and 300 bp MiSeq PET reads for assembly, and was scaffolded with the MPET and Moleculo (a.k.a. TruSeq) synthetic long reads (Illumina, San Diego, CA). The addition of the 250 bp HiSeq PET reads from 4 new sequencing libraries nearly doubled the sequence coverage of

the genome, and yielded a new base assembly. This assembly was then scaffolded with the MPET and Moleculo reads, as well as the BART reference transcriptome and another ABySS assembly generated at a lower k value, to yield version 2, which is available from NCBI under accession LIAG00000000. The gene annotation, comparative genomics, and differential expression experiment were performed on version 2 of the genome sequence, as indicated in the manuscript. The version 3 assembly was produced by rescaffolding the version 2 assembly using Chromium linked reads from 10X Genomics (Pleasanton, CA) and the ARCS scaffolding software developed by our group. This assembly has been submitted to NCBI, and early access to it and its annotations are available on the BCGSC ftp site at ftp://ftp.bcgsc.ca/supplementary/bullfrog.

TH experiment

We sequenced transcriptomes from the back skin of three individual *R. catesbeiana* tadpoles that were injected with 10 pmol/g body weight of T3 (Sigma-Aldrich Canada Ltd.) prepared in dilute NaOH (ACP Chemicals Inc.) and sacrificed 48 h post-injection. A matched group of vehicle only-injected tadpoles consisted of an additional group of 3 individual animals. Details of the exposures and evidence of tissue responsiveness to T3 treatment using qPCR of these animals can be found in Maher *et al.* (2016). These samples were also used by Maher *et al.* (2016), but within the context of a separate study with distinct analyses focused solely on targeted qPCR of select mRNA transcripts. The samples were randomized during processing and the technician was blind to the hormone treatment status.

Single-stranded RNA-Seq libraries were generated from these six samples individually using Illumina HiSeq 2500 paired-end sequencing platform (San Diego, CA, USA) and 100 base pair (bp) paired end sequencing protocol following manufacturer's instructions. Information on the six

read libraries is shown in Supplementary Table 5. The high read depth per library at this sample size is expected to yield adequate statistical power for the differential expression analysis[14].

qPCR analysis of transcript abundance

Transcript abundance of select transcripts encoding proteins involved in RNA/DNA processing and lncRNAs was determined using methods and conditions published previously[15]. The primer sequences, annealing temperatures, and amplicon sizes are shown in Supplementary Table 4.

lncRNA detection

The workflow used to detect candidate lncRNAs is summarized in Supplementary Figure 9. First, open reading frames (ORFs) were predicted using TransDecoder v3.0.0 (transdecoder.github.io) with the default parameters, and contigs with complete or partial predicted ORFs were excluded. We also performed 3-frame *in silico* translations of the contigs to evaluate the validity of any potential encoded peptides via comparison to the Pfam curated database of peptide motifs[16] using HMMScan v3.1b2 from the HMMER package[17]. Furthermore, we did a six-frame translation of our nucleotide sequences, and queried them against Uniref90[18] and NCBI's RefSeq databases using the BLASTx program from NCBI's BLAST+ (v2.4.0) software package[2]. We discarded all contigs that returned a hit to any sequence in these databases at e-value < 10^{-5}. We constructed a comprehensive amphibian transcriptome shotgun assembly database (CATSA) by downloading and combining nucleotide sequences for 16 amphibian species (Supplementary Table 4) from the NCBI Genbank Transcriptome Shotgun Assembly Sequence (TSA) database[19]. We interrogated our putative lncRNA contig set against this CATSA database for homologs that could add confidence to our set. We also did a similarity search against lncRNA sequences present in lncRNADB[20] and LNCipedia[21], which are databases of previously reported lncRNAs.

We assessed the coding potential of our contigs with Coding Potential Calculator (CPC)[22] v0.9-r2, and filtered out any contig that returned a CPC score greater than 1.

Repetitive sequence element detection

The content of repetitive sequence elements in the version 2 draft genome assembly was evaluated with RepeatMasker[4] (version 4.0.6) with default settings. The RepBase collection of repeat sequence elements was supplemented with novel elements identified using RepeatModeler[23] (version 1.0.8) with RMBlast (version 2.2.27+, http://www.repeatmasker.org/RMBlast.html) applied to the draft genome assembly with default settings.

Supplementary References

1 Kucuk, E. *et al.* Kollector: transcript-informed, targeted *de novo* assembly of gene loci. *Bioinformatics* **33,** 1782-1788 (2017).

2 Camacho, C. *et al.* BLAST+: architecture and applications. *BMC Bioinformatics* **10,** 421 (2009).

3 Holt, C. & Yandell, M. MAKER2: an annotation pipeline and genome-database management tool for second-generation genome projects. *BMC Bioinformatics* **12,** 491 (2011).

4 Smit, A. F. A., Hubley, R. & Green, P. *RepeatMasker Open-4.0*, <http://www.repeatmasker.org/> (2015).

5 Jurka, J. *et al.* Repbase Update, a database of eukaryotic repetitive elements. *Cytogenet. Genome Res.* **110,** 462-467 (2005).

6 Stanke, M., Schoffmann, O., Morgenstern, B. & Waack, S. Gene prediction in eukaryotes with a generalized hidden Markov model that uses hints from external sources. *BMC bioinformatics* **7,** 62 (2006).

7 Korf, I. Gene finding in novel genomes. *BMC Bioinformatics* **5,** 59 (2004).

8 Ter-Hovhannisyan, V., Lomsadze, A., Chernoff, Y. O. & Borodovsky, M. Gene prediction in novel fungal genomes using an *ab initio* algorithm with unsupervised training. *Genome Res.* **18,** 1979-1990 (2008).

9 Slater, G. S. & Birney, E. Automated generation of heuristics for biological sequence comparison. *BMC Bioinformatics* **6,** 31 (2005).

10 UniProt, C. UniProt: a hub for protein information. *Nucleic Acids Res.* **43,** D204-212 (2015).

11 Cantarel, B. L. *et al.* MAKER: an easy-to-use annotation pipeline designed for emerging model organism genomes. *Genome Res.* **18,** 188-196 (2008).

12 Huang, D. W., Sherman, B. T. & Lempicki, R. A. Systematic and integrative analysis of large gene lists using DAVID bioinformatics resources. *Nat. Protoc.* **4,** 44-57 (2009).

13 Supek, F., Bošnjak, M., Škunca, N. & Šmuc, T. REVIGO summarizes and visualizes long lists of gene ontology terms. *PloS ONE* **6,** e21800 (2011).

14 Ching, T., Huang, S. & Garmire, L. X. Power analysis and sample size estimation for RNA-Seq differential expression. *RNA* **20,** 1684-1696 (2014).

15 Maher, S. K. *et al.* Rethinking the biological relationships of the thyroid hormones, l-thyroxine and 3,5,3'-triiodothyronine. *Comp. Biochem. Physiol. Part D Genomics Proteomics* **18,** 44-53 (2016).

16 Finn, R. D. *et al.* Pfam: the protein families database. *Nucleic Acids Res.* **42,** D222-230 (2014).

17 Finn, R. D. *et al.* HMMER web server: 2015 update. *Nucleic Acids Res.* **43,** W30-38 (2015).

18 Suzek, B. E. *et al.* UniRef clusters: a comprehensive and scalable alternative for improving sequence similarity searches. *Bioinformatics* **31,** 926-932 (2015).

19 Clark, K., Karsch-Mizrachi, I., Lipman, D. J., Ostell, J. & Sayers, E. W. GenBank. *Nucleic Acids Res.* **44**, D67-72 (2016).

20 Quek, X. C. *et al.* lncRNAdb v2.0: expanding the reference database for functional long noncoding RNAs. *Nucleic Acids Res.* **43,** D168-173 (2015).

21 Volders, P. J. *et al.* An update on LNCipedia: a database for annotated human lncRNA sequences. *Nucleic Acids Res.* **43,** D174-180 (2015).

22 Kong, L. *et al.* CPC: assess the protein-coding potential of transcripts using sequence features and support vector machine. *Nucleic Acids Res.* **35,** W345-349 (2007).

23 Smit, A. F. A. & Hubley, R. *RepeatModeler Open-1.0*, <http://www.repeatmasker.org> (2015).

24 Tamura, K. & Nei, M. Estimation of the number of nucleotide substitutions in the control region of mitochondrial DNA in humans and chimpanzees. *Mol. Biol. Evol.* **10,** 512-526 (1993).

25 Kumar, S., Stecher, G. & Tamura, K. MEGA7: molecular evolutionary genetics analysis version 7.0 for bigger datasets. *Mol. Biol. Evol.* **33,** 1870-1874 (2016).

26 Sun, Y. B. *et al.* Whole-genome sequence of the Tibetan frog *Nanorana parkeri* and the comparative evolution of tetrapod genomes. *Proc. Natl. Acad. Sci. USA* **112,** E1257-1262 (2015).

27 Beaudoing, E., Freier, S., Wyatt, J. R., Claverie, J. M. & Gautheret, D. Patterns of variant polyadenylation signal usage in human genes. *Genome Res.* **10,** 1001-1010 (2000).
